# The Combination of Baicalein and Memantine Reduces Oxidative Stress and Protects against β-amyloid-Induced Alzheimer’s Disease in Rat Model

**DOI:** 10.3390/antiox12030707

**Published:** 2023-03-13

**Authors:** Ratnakar Jadhav, Yogesh A. Kulkarni

**Affiliations:** Shobhaben Pratapbhai Patel School of Pharmacy & Technology Management, SVKM’s NMIMS, V.L. Mehta Road, Vile Parle (West), Mumbai 400056, India

**Keywords:** baicalein, β-amyloid, BDNF, oxidative stress, neurodegeneration, Alzheimer’s disease

## Abstract

Alzheimer’s disease (AD) is a neuronal condition causing progressive loss of memory and cognitive dysfunction particularly in elders. An upsurge in the global old age population has led to a proportionate increase in the prevalence of AD. The current treatments for AD are symptomatic and have debilitating side effects. A literature review and current research have directed scientists to explore natural products with better safety and efficacy profiles as new treatment options for AD. Baicalein, belonging to the flavone subclass of flavonoids, has been reported for its anti-oxidant, anti-inflammatory, AChE enzyme inhibitory activity and anti-amyloid protein aggregation activity, which collectively demonstrates its benefits as a neuroprotective agent. Presently, memantine, a NMDAR antagonist, is one of the important drugs used for treatment of Alzheimer’s disease. The current study aims to investigate the effect of baicalein in combination with memantine in β-amyloid-induced AD in albino Wistar rats. Baicalein (10 mg/kg) alone, 5 mg/kg and 10 mg/kg in combination with memantine (20 mg/kg) was administered for 21 days. Treatment with baicalein in combination with memantine showed significant improvement in behavioural studies. The combination treatment decreased oxidative stress, β-amyloid plaque formation and increased the expression of brain-derived neurotrophic factor (BDNF) in the brain. From the results, it can be concluded that treatment with baicalein and memantine could be beneficial for reducing the progression of neurodegeneration in rats. Baicalein has an additive effect in combination with memantine, making it a potential option for the treatment of AD.

## 1. Introduction

Alzheimer’s disease (AD) is a type of dementia affecting memory, thinking and behavior. The majority of the dementia cases belong to AD—a progressive neurodegenerative disorder of the aging population. AD becoming a major burden on the global healthcare system. With the access to healthcare facilities and increasing life expectancy, the number of people living over 65 years of age is rapidly growing. In 2019, there were 703 million people over the age of 65 years, which is predicted to double by 2050 [1]. This increase in the aging population can have a significant impact on the social and healthcare sectors. It has been reported that dementia is the 7th leading cause of mortality and has the highest costs to society. The estimated global direct cost of dementia care will be $2 trillion by 2030, and will increase to $9.12 trillion by 2050 [2]. Dementia has created an unmet need to scale up investment in research related to dementia care and treatment. 

The distinguishing characteristics of AD are extracellular β-amyloid deposits and intracellular neurofibrillary tangles (NFT) in the brain to the extent that it impacts memory and cognitive functions [3]. It is understood that the neurotoxic β-amyloid plaques deposited in the hippocampus and basal brain attracts more insoluble neurotoxic β-amyloid plaques, inducing mitochondrial damage and synaptic dysfunction [4]. Activation of microglia and astrocytes stimulates inflammatory signals and increases reactive oxygen species (ROS), promoting the destruction of mitochondrial macromolecules [5]. Moreover, β-amyloid has been reported to reduce the activity of superoxide dismutase (SOD), an enzyme that scavenges superoxide radicals and prevents peroxidative damage. β-amyloid also interacts with Aβ-binding alcohol dehydrogenase (ABAD), inhibiting its activity and causing accumulation of lipid peroxides (LPO) and 4-Hydroxy-2-nonenal (4-HNE), resulting in neuronal death [6]. A loss of homeostasis due to excessive levels of cellular ROS or a decrease in the variety of oxidative defense mechanism causes damage to proteins, nucleic acids, lipids, and membranes, resulting in neuronal cell death by necrosis or apoptosis [7,8]. 

Increased oxidative stress contributes to basal forebrain cholinergic neuron degeneration. This impairment in cholinergic neurons results in cognitive dysfunction. Studies have revealed that inhibition of acetylcholine esterase enhanced neural functions by increasing ACh levels [9]. Brain-derived neurotrophic factor (BDNF) has an important role in maintaining synaptic plasticity which is central to learning and memory dysfunction. However, β-amyloid disrupts BDNF signalling through dysregulation of the N-methyl-D-aspartate receptor (NMDAR) signalling cascade [10].

The available treatment for AD is cholinesterase inhibitors (CIs), i.e., donepezil and rivastigmine for mild to moderate symptoms and memantine, an NMDA receptor antagonist, for moderate to severe symptoms. Memantine hydrochloride (3,5-Dimethyl-1-adamantanamine hydrochloride) is a low-affinity, non-competitive NMDA receptor antagonist. Memantine only inhibits the extensive NMDA receptor activity; hence, it does not hinder the normal synaptic transmission and physiological activity. It is understood that memantine blocks the permeability of Ca^2+^ which has diverse effect on the long-term depression or potentiation, leading to neuronal cell death. The low to moderate affinity for the receptor is critical for the pharmacological activity of memantine. Memantine inhibits the excessive activity of glutamate while enabling the activation of NMDARs required for cognitive functions and memory [11]. 

In 2014, the FDA approved the combination of donepezil and memantine for the management of moderate to severe symptoms of AD. The literature has reported serious gastrointestinal and neurological adverse effects with cholinesterase inhibitors, and hence, they may require dose adjustment [12]. As the current treatment does not cure the disease but only provides symptomatic relief with major side effects, there is a mandate for new therapies to treat AD. The present research has shed light on the expediency for combination therapies of pharmaceuticals with natural products. 

Products of natural origin have been used for decades due to their safe and effective profile in treating various diseases. In recent years, scientists have been interested in flavonoids, one of the major groups of natural products, reported to have various therapeutic properties, such as anti-oxidant, anti-inflammatory, anti-cancer, cardio-protective, hepato-protective, and neuro-protective effects [13,14].

Baicalein (5,6,7-trihydroxyflavone 7-O-beta-D-glucuronide) is an isoflavone extracted from the roots of *Scutellaria baicalensis Georgi* (Labiatae). It has gained attention for its various pharmacological effects, including anti-oxidant, anti-viral, anti-cancer, anti-inflammatory, cardio-protective, acetyl-cholinesterase-inhibitory, monoamine-oxidase (MAO)-inhibitory and neuro-protective activity. Baicalein also permeates the blood brain barrier within 20–30 min of administration [15,16,17,18,19].

Although, the pharmacological properties of baicalein have been shown in many in vitro and in vivo studies, its therapeutic use as an alternative or in combination with currently approved treatments for AD has not been fully elucidated in any of the animal studies conducted till date. Hence, the current study provides insight into the therapeutic potential of baicalein in combination with memantine on β-amyloid-induced AD in albino Wistar rats. The dose of the baicalein used in this study was based on the previously published studies of baicalein.

## 2. Material and Methods

### 2.1. Drugs and Chemicals

Memantine (100.1% *w*/*w* HPLC assay) was received from Intas Pharmaceuticals (India) as a gift sample. Baicalein (98% pure) was procured from Chemical Centre (Mumbai, India). β-amyloid peptide and acetylcholine iodide were purchased from Sigma Aldrich, Rockville, MD, USA. β-amyloid antibody (Catalog No.: SC-28365 Lot# I0117) and BDNF antibody (Catalog No.: SC-65514, Lot# L0816) were purchased from Santacruz Biotechnology Inc., (Dallas, TX, USA). 

### 2.2. Experimental Animals 

Male albino Wistar rats (180–200 g) were obtained from the National Institute of Biosciences (NIB), Pune, India. Animals were accustomed to the laboratory conditions for a week before the start of the experiment. They also had access to standard diet and water ad libitum throughout the experiment. The animal study was reviewed and approved by the Institutional Animal Ethics Committee of Shri Vile Parle Kelavani Mandal, Mumbai. The experimental procedures were performed in compliance with the norms of the Committee for the Purpose of Control and Supervision of Experiments on Animals (CPCSEA), Government of India. 

### 2.3. Experimental Design 

Based on the body weight, albino Wistar rats (180–220 g) were randomized into six groups: (1) normal control group (received 0.9% NaCl); (2) disease control group (receiving 400 pmol Aβ_(1–42)_ peptide); (3) memantine (received 20 mg/kg orally); (4) baicalein (10 mg/kg orally); (5) memantine and baicalein (20 + 5 mg/kg orally); and (6) memantine and baicalein (20 + 10 mg/kg orally).

All the animals underwent stereotaxic surgery and were administered an intra-cerebro–ventricular (i.c.v.) injection of 400 pmol of Aβ_(1–42)_, except animals from the normal control group, which were administered normal saline (0.9% NaCl) [20].

Aβ_(1–42)_ peptide was prepared by dissolving it in distilled water and pre-aggregated by incubating at 37 °C for four days before use [21]. Then, stereotaxic surgery was performed under anesthesia. The dose of baicalein was decided on based on the published literature [22]. The treatment was started after 21 days of stereotaxic surgery to ensure the development of neurodegeneration and continued for next 21 days.

### 2.4. Surgical Procedures

The below-mentioned surgical process was followed [23]. Ketamine (80 mg/kg, *i.p.*) and xylazine (10 mg/kg, *i.m.*) were used to anaesthetize animals before the surgery. The head was shaved and disinfected with an iodine tincture. The animal was mounted on a stereotaxic apparatus. A vertical cut on the head skin using sterile scissors was made along the median longitudinal calvaria. The bregma was identified and, using a bone drill, a hole with a 1 mm diameter was drilled approximately 2 mm posterior (P) and 1.4 mm lateral (L) in the antero-dorsal thalamic nucleus for injecting Aβ_(1–42)_ peptide. The surface of the skull was cleaned repeatedly with sterile dry cotton. A total of 4 μL of Aβ_(1–42)_ peptide was injected at a rate of 1 μL/min using a micro syringe (Hamilton^®^) attached to a stainless-steel cannula through a polypropene (PP) tube. The cannula was kept inside the brain for 5 min and then slowly removed. Using bone cement, the hole in the skull was sealed. The incision was closed by suturing and applying an iodine tincture and Neosporin^®^ dusting powder. After the surgery, each animal was kept in a separate cage. Every day, an iodine tincture and Neosporin^®^ dusting powder were applied on the wound until it was healed.

### 2.5. Behavioural Assessment

#### 2.5.1. Locomotor Activity

A digital Actophotometer (Inco, India) was used to assess the movement of each animal on day 21 and 42 of injecting Aβ_(1–42)_ [24]. Each animal was placed in the Actophotometer for 5 min and was observed for its locomotor activity. The Actophotometer was equipped with infrared light-sensitive photocells. The movement of the animal within the compartment disturbs the light falling on the photocells mounted on the opposite wall, which was noted by the instrument.

#### 2.5.2. Morris Water Maze (MWM) Test

This test was conducted as per methods previously reported in the literature [25]. The MWM comprised a round swimming tank of 150 cm diameter and 30 cm depth. The round maze was divided into four equal quadrants by marking the edges with tape. A sturdy platform was placed in one of the quadrants. Each animal was exposed to MWM during two phases: an acquisition phase on day 17–20 and a retention phase on day 21 and 42. During the acquisition phase, each animal was subjected to 4 trials with a 10 min gap; each time, it was placed in a different quadrant facing the wall. The animal was allowed to explore the tank and find the platform for 120 s. If the animal was unable to find the platform, it was directed towards it and permitted to stay on it for an amount of time. During the acquisition phase, the platform was visible to the animal, as the water level was kept 1 cm below the surface of the platform. However, during the retention phase, water in the tank was made opaque and the platform surface was not visible to the animal, as water level was 1 cm above the surface of the platform. During both phases, the time required for the animal to reach the platform was recorded. 

#### 2.5.3. Elevated Plus Maze (EPM) Test

This test was executed as per the method published previously in the literature [26]. The EPM had four arms: two open and two closed, each connected with a central square. The dimensions of the arms were 50 cm in length, 10 cm in width, and placed at a height of 50 cm from the floor. The test was carried out in two phases: an acquisition phase, where initial transfer latency (ITL) was recorded; and a retention phase, where the 1st and 2nd transfer latencies were recorded. The acquisition phase was performed on day 20 while the retention phase was conducted on days 21 and 42. During the experiment, the animal was placed in the open arm facing outward from the central square. The transfer latency was recorded as the time required for the animal to go inside the closed arm. 

#### 2.5.4. Passive Avoidance (PA) Test

Passive Avoidance (PA) equipment comprises two compartments separated by a collapsible door. One compartment was kept dark while the other was lit with a light bulb. The floor of the dark compartment was made up of metal mesh attached to an electric supply for applying an electric shock (40 V, 0.5 mA for 2 s). Each animal was exposed to the apparatus for two trials: the acquisition trail, which was performed on day 20, and the retention trial, which was performed on days 21 and 42 [27,28]. The animal was kept in the lit compartment and was permitted to explore the compartment for 60 s. Then, the door separating the two compartments was opened. As the animal enters the dark compartment, an electric shock was given and the animal moved to its home cage. The amount of time required for the animal enter into the dark compartment after opening the door was recorded as pre-shock latency. The experiment was repeated on day 21 and 42 to record the post-shock latency.

### 2.6. Biochemical Assessment

The rats were sacrificed by CO_2_ asphyxiation, and brain tissues were removed. The cortex and hippocampus were separated to perform various biochemical and histopathological evaluations.

#### 2.6.1. Measurement of Oxidative Stress Parameters

The oxidative stress parameters, including malondialdehyde (MDA), superoxide dismutase (SOD), catalase and reduced glutathione (GSH) were estimated in the hippocampus and cortex.

The cortex and hippocampus harvested from experimental animals and homogenized in an ice-cold phosphate buffer (0.1 M, pH 7.4) using probe homogenizer (Polytron PT 2500E, Kinematica, Malters, Switzerland). MDA was calculated as per the process described by Ohkawa et al. [29]. SOD was estimated as per the method defined by Paoletti et al. [30], the determination of catalase was carried out based on the procedure defined by Luck et al. [31] and the assessment of GSH was performed as per the procedure of Ellman et al. [32]. 

#### 2.6.2. Measurement of Acetyl Cholinesterase Activity

The assessment of the acetyl cholinesterase activity was carried out based on the previously published procedure [33]. 

### 2.7. Histopathological Assessment

Formalin-fixed brain tissue samples were used for histopathological studies. The hippocampus and cortex were evaluated separately for the histopathological indicators. These tissues were trimmed and underwent a dehydration process of ascending alcohol concentrations, followed by clearing through xylene and an embedding in paraffin wax. The Rotary Microtome was used to section paraffin-embedded tissue blocks to a thickness of 4–6 µm. The sections were stained with H&E and Congo red for the assessment of pathological lesions and neuronal degeneration under the photographic microscope at 400×.

### 2.8. Immunohistochemistry Assessment (IHC)

Immunohistochemical (IHC) assessment for the expression of Aβ and BDNF in both hippocampus and cortex was performed. Paraffin-wax-embedded tissue blocks were placed in microtome for taking sections of 4–6 µm thickness and fixed on slides coated with Poly-L-Lysine and incubated overnight at 4 °C. These sections were deparaffinized, rehydrated and incubated with citrate buffer at pH 6 in the decloaking chamber. Slides were incubated in a 3% hydrogen peroxide block for 20 min to inhibit endogenous peroxidase. For the assessment of Aβ expression, the primary antibody was a β-Amyloid (b-4) antibody, and the secondary antibody was peroxidase-labeled anti-rabbit IgG. The staining was visualized by a reaction with a diaminobenzidine color reagent and then counterstained with hematoxylin. Finally, the sections were rinsed with Tris-buffered saline (TBS), dehydrated in alcohol and cleared in xylene before mounting using DPX. All the sections were examined under a light microscope to record the intensity of the antigen–antibody reaction.

For the assessment of BDNF expression, pro-BDNF-5H8 antibodies were used as the primary antibody, whereas anti-rabbit-IgG was used as the secondary antibody for immunoreaction.

The evaluation of the IHC assessment was performed based on the microscopic observations. The classification of immunoreactivity was performed based on the % of positive neuronal cells, their distribution and intensity of staining. The 0% positive cells were considered as normal, <1% as minimal, 1–25% as mild, 26–50% as moderate, 51–75% as marked/moderately severe and 76–100% as severe in terms of staining. Distribution was recorded as focal, multifocal and diffuse.

### 2.9. Statistical Analysis

GraphPad Prism V8.0 was used for the statistical analysis of all the parameters. Two-way ANOVA (analysis of variance) and Bonferroni’s test at a level of significance of *p* < 0.05 were used for the evaluation of behavioural parameters. The oxidative stress parameters, AChE activity and optical density data for IHC studies were evaluated using one-way ANOVA using Dunnett’s test at a level of significance of *p* < 0.05. The optical density (OD) data was evaluated using ImageJ 1.8.0 software. 

## 3. Results

### 3.1. Behavioural Assessment

#### 3.1.1. Locomotor Activity

**Figure 1:**
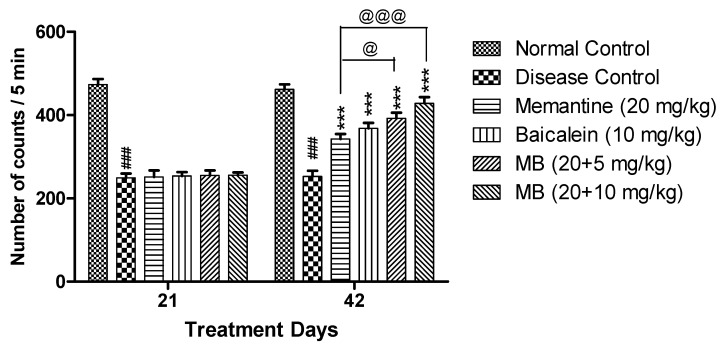
Actophotometer—The locomotor activity of each treatment group was plotted against treatment days (21 and 42 days). Data are shown as mean ± SEM (n = 6) at *p* < 0.05. Comparison with normal control is indicated with ### *p* < 0.001, whereas comparison with disease control is indicated with *** *p* < 0.001; @ *p* < 0.05 and @@@ *p* < 0.001 indicate comparison with memantine group. MB: memantine + baicalein.

The disease control group displayed reduced locomotor activity as compared to normal control group. The locomotor activity was significantly increased on day 42 (*p* < 0.001) in both the treatment groups of baicalein with memantine when compared with the disease control group. There was significant increase in locomotor activity in animals treated with baicalein with memantine when compared with animals treated with memantine (20 mg/kg) alone (Figure 1).

#### 3.1.2. Morris Water Maze Test

**Figure 2:**
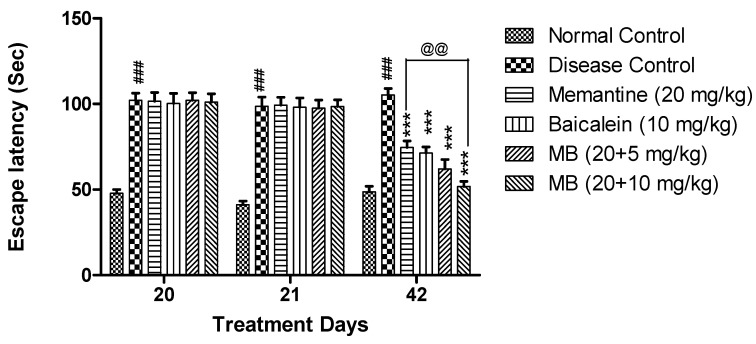
Morris water maze—The escape latency of each treatment group was plotted against treatment days (20, 21 and 42 days). Data are shown as mean ± SEM (n = 6) at *p* < 0.05. Comparison with normal control is indicated with ### *p* < 0.001, whereas comparison with disease control is indicated with *** *p* < 0.001 and @@ *p* < 0.01 indicate comparison with memantine treatment group. MB: memantine + baicalein.

The disease control group exhibited significantly increased (*p* < 0.001) escape latency when compared with the normal control group. The escape latency was significantly reduced (*p* < 0.001) in animals treated with memantine and baicalein at doses of 20 + 5 and 20 + 10 mg/kg when compared with the disease control group. Moreover, the escape latency was also significantly reduced (*p* < 0.01) in animals treated with memantine and baicalein at a dose of 20 + 10 mg/kg when compared with animals treated with memantine (20 mg/kg) alone (Figure 2).

#### 3.1.3. Elevated Plus Maze Test

**Figure 3:**
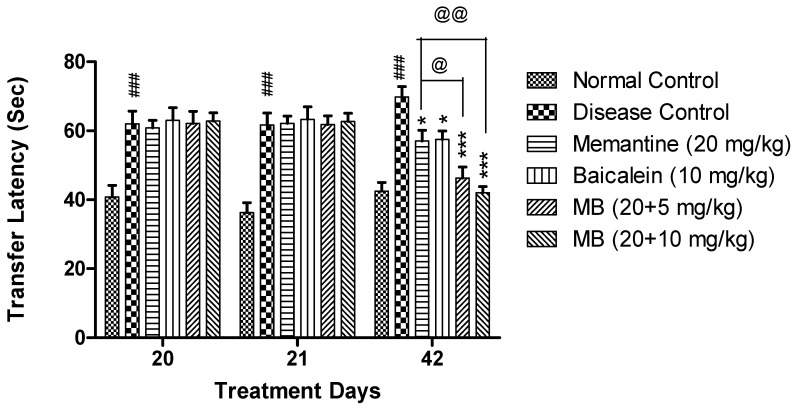
Elevated Plus maze—The transfer latency of each treatment group was plotted against treatment days (20, 21 and 42 days). Data are shown as mean ± SEM (n = 6) at *p* < 0.05. Comparison with normal control is indicated with ### *p* < 0.001, whereas comparison with disease control is indicated with * *p* < 0.05, *** *p* < 0.001, @ *p* < 0.05 and @@ *p* < 0.01 indicate comparison with memantine treatment group. MB: memantine + baicalein.

In EPM, on both days 21 and 42, the transfer latency in the disease control group was significantly increased (*p* < 0.001) as compared to the normal control group. The combination of baicalein with memantine at both the doses of 20 + 5 and 20 + 10 mg/kg showed significantly reduced transfer latency as compared to the disease control group. Moreover, at the dose of 20 + 10 mg/kg, the combination treatment of baicalein with memantine decreased (*p* < 0.01) the transfer latency when compared with memantine (20 mg/kg) alone (Figure 3). 

#### 3.1.4. Passive Avoidance Test

**Figure 4:**
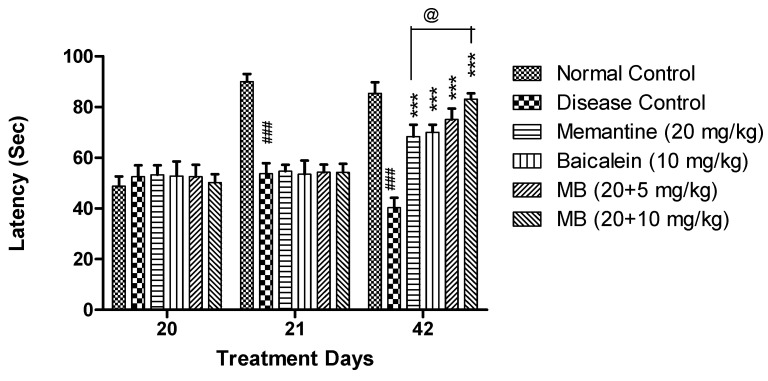
Passive Avoidance—The post shock latency of each treatment group was plotted against treatment days (20, 21 and 42 days). Data are shown as mean ± SEM (n = 6) at *p* < 0.05. Comparison with normal control is indicated with ### *p* < 0.001 whereas comparison with disease control is indicated with *** *p* < 0.001 and @ *p* < 0.05 indicate comparison with memantine treatment group. MB: memantine + baicalein.

In PA, on day 21 and 42, a significant decrease (*p* < 0.001) in post-shock latency was observed in the disease control group when compared with the normal control group. The combination treatment of baicalein with memantine at both the doses of 20 + 5 mg/kg and 20 + 10 mg/kg showed a significant increase in post-shock latency (*p* < 0.001) as compared to the disease control group on day 42. Moreover, the treatment of baicalein with memantine (20 + 10 mg/kg) increased (*p* < 0.05) post-shock latency when compared with the animals treated with memantine (20 mg/kg) alone (Figure 4).

### 3.2. Effect of Oxidative Stress Parameters 

Compared with the normal control group, a significant increase in MDA levels (*p* < 0.001) was observed in both the hippocampus and cortex of the disease control group, while a substantial reduction (*p* < 0.001) in the levels of SOD, catalase and GSH were observed. The hippocampus and cortex of animals treated with baicalein and memantine at the dose of 20 + 10 mg/kg showed significant decline in the levels of MDA with an increase in the levels of SOD, catalase and GSH as compared to the disease control group. Furthermore, the combination treatment at the dose of 20 + 10 mg/kg showed a significant effect when compared with the memantine (20 mg/kg) alone treatment group, indicating an additive effect in the management of oxidative stress induced by β-amyloid in the rat brain (Table 1 and Table 2).

### 3.3. Acetyl Cholinesterase Activity 

**Figure 5:**
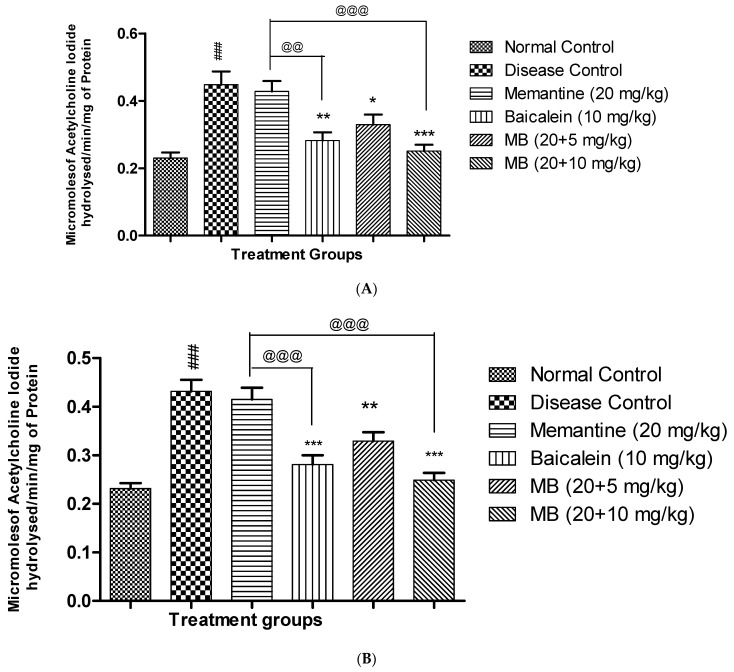
(**A**). Effect of Aβ_(1–42)_, baicalein and baicalein with memantine on AChE in the rat brain hippocampus. Data are represented as mean ± SEM (n = 6), where ### *p* < 0.001 indicates comparison with normal control, * *p* < 0.05, ** *p* < 0.01 and *** *p* < 0.001 indicates comparison with disease control group, whereas @@ *p* < 0.01 and @@@ *p* < 0.001 indicates comparison with memantine (20 mg/kg) group. SEM: Standard Error of Mean. (**B**). Effect of Aβ_(1–42)_, baicalein and baicalein with memantine on AChE in the rat brain cortex.

The AChE activity in the hippocampus and cortex of animals from the disease control group was significantly higher (*p* < 0.001) than the normal control group. Treatment of baicalein with memantine at a dose of 20 + 10 mg significantly reduced (*p* < 0.001) AChE activity in both the hippocampus and cortex region when compared with the disease control group. The combination treatment at a dose of 20 + 10 mg showed a significant AChE-inhibitory effect as compared to memantine (20 mg/kg) alone in the hippocampus and cortex (Figure 5A,B).

### 3.4. Histopathological Study

#### 3.4.1. Haematoxylin and Eosin Staining

**Figure 6:**
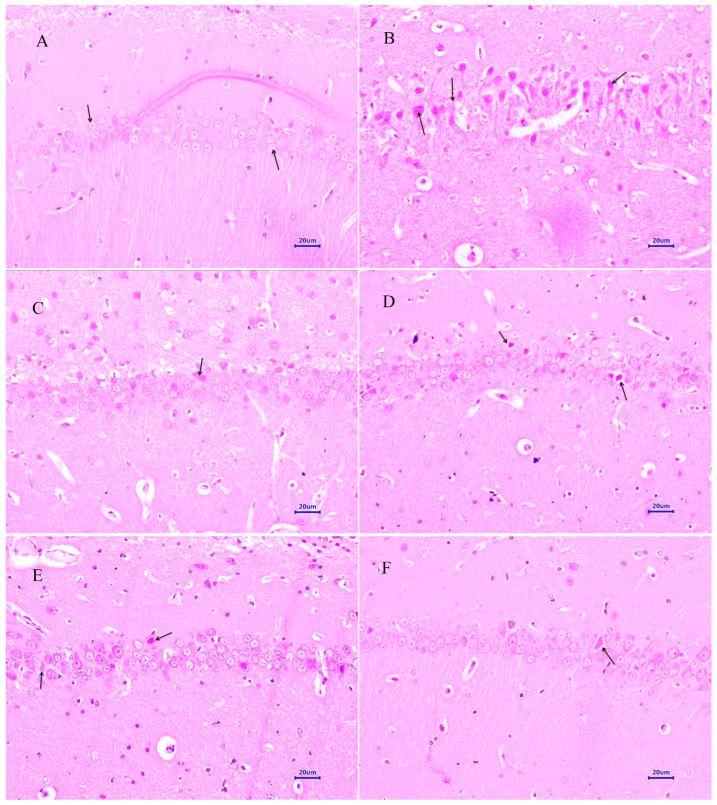
Effect of Aβ_(1–42),_ baicalein and baicalein with memantine on H&E-stained hippocampus tissue (400×, scale bar = 20 µm). (**A**) Normal control: displaying normal histology, normal neuronal cells in hippocampus (arrow). (**B**) Disease control: displaying multifocal reduced layer of neuronal cells (arrow) and marked neurodegeneration with pyknotic nuclei (arrow) in hippocampus. (**C**) Memantine 20 mg/kg: displaying mild to moderate neurodegeneration with pyknotic nuclei in hippocampus (arrow). (**D**) Baicalein (10 mg/kg): displaying multifocal minimal to mild neurodegeneration with pyknotic nuclei in hippocampus (arrow). (**E**) Memantine + Baicalein (20 + 5 mg/kg): displaying multifocal minimal to mild neurodegeneration with pyknotic nuclei in hippocampus (arrow). (**F**) Memantine + Baicalein (20 + 10 mg/kg): displaying mild neurodegeneration with pyknotic nuclei in hippocampus (arrow).

**Figure 7:**
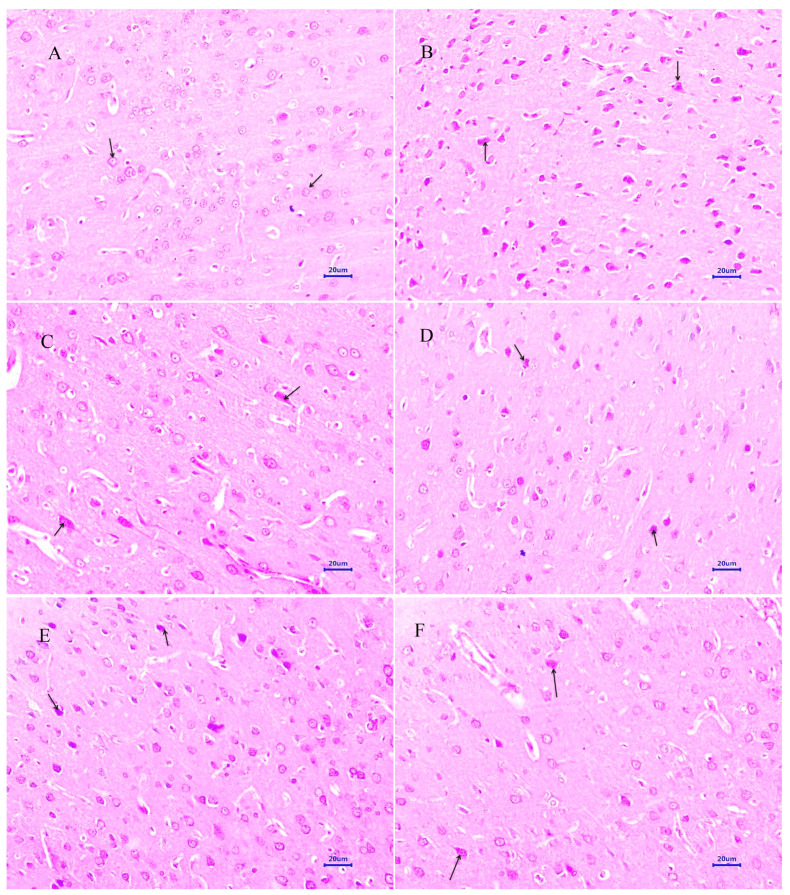
Effect of Aβ_(1–42),_ baicalein and baicalein with memantine on H&E-stained cortex tissue (400×). (**A**) Normal control: displaying normal histology, normal neuronal cells in parietal cortex (arrow). (**B**) Disease control: displaying multifocal reduced layer of neuronal cells and sever neurodegeneration with pyknotic nuclei in cortex (arrow). (**C**) Memantine 20 mg/kg: displaying moderate neurodegeneration with pyknotic nuclei in cortex (arrow). (**D**) Baicalein (10 mg/kg): displaying multifocal mild to moderate neuronal degeneration with pyknotic nuclei in cortex (arrow). (**E**) Memantine + Baicalein (20 + 5 mg/kg): displaying multifocal minimal to mild neurodegeneration with pyknotic nuclei in cortex (arrow). (**F**) Memantine + Baicalein (20 + 10 mg/kg): displaying minimal neuronal degeneration in cortex (arrow).

Microscopic examination of the hippocampus and cortex sections from animals from the normal control group did not reveal any signs of pathological significance. However, animals from the disease control group showed multi-focal moderate to marked neurodegeneration in the hippocampus and cortex. Animals treated with memantine 20 mg/kg showed a focal mild to moderate reduced layer of neuronal cells in the hippocampus, whereas examination of the cortex exhibited multi-focal mild to moderate neuronal degeneration. Treatment of baicalein (10 mg/kg) demonstrated multifocal minimal to mild neurodegeneration in both the hippocampus and cortex. Moreover, combination treatment of baicalein with memantine at the dose of 20 + 5 mg/kg also revealed multifocal minimal to mild neuronal degeneration in the hippocampus and cortex. Furthermore, the treatment of baicalein and memantine at the dose of 20 + 10 mg/kg demonstrated an additive effect with focal minimal to mild neurodegeneration in both hippocampus and cortex regions of the rat brain (Figure 6 and Figure 7).

#### 3.4.2. Congo Red Staining

**Figure 8:**
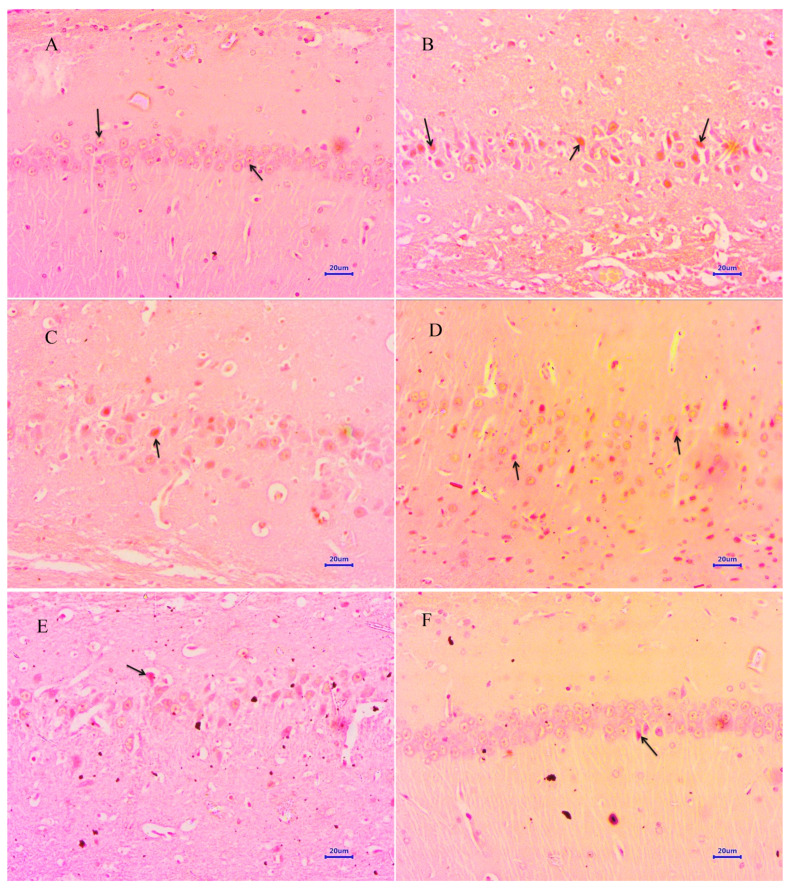
Effect of Aβ_(1–42),_ baicalein and baicalein with memantine on Congo-red-stained hippocampus (400×). (**A**) Normal control: displaying normal histology, normal neuronal cells in hippocampus (arrow). (**B**) Disease control: displaying multifocal moderate to severe deposition of β-amyloid (arrow) in hippocampus. (**C**) Memantine 20 mg/kg: displaying multifocal moderate deposition of β-amyloid (arrow) in hippocampus. (**D**) Baicalein (10 mg/kg): displaying multifocal mild to moderate deposition of β-amyloid deposition (arrow) in hippocampus. (**E**) Memantine + Baicalein (20 + 5 mg/kg): displaying multifocal mild deposition of β-amyloid (arrow) in hippocampus. (**F**) Memantine + Baicalein (20 + 10 mg/kg): displaying focal mild deposition of β-amyloid (arrow) in hippocampus.

**Figure 9:**
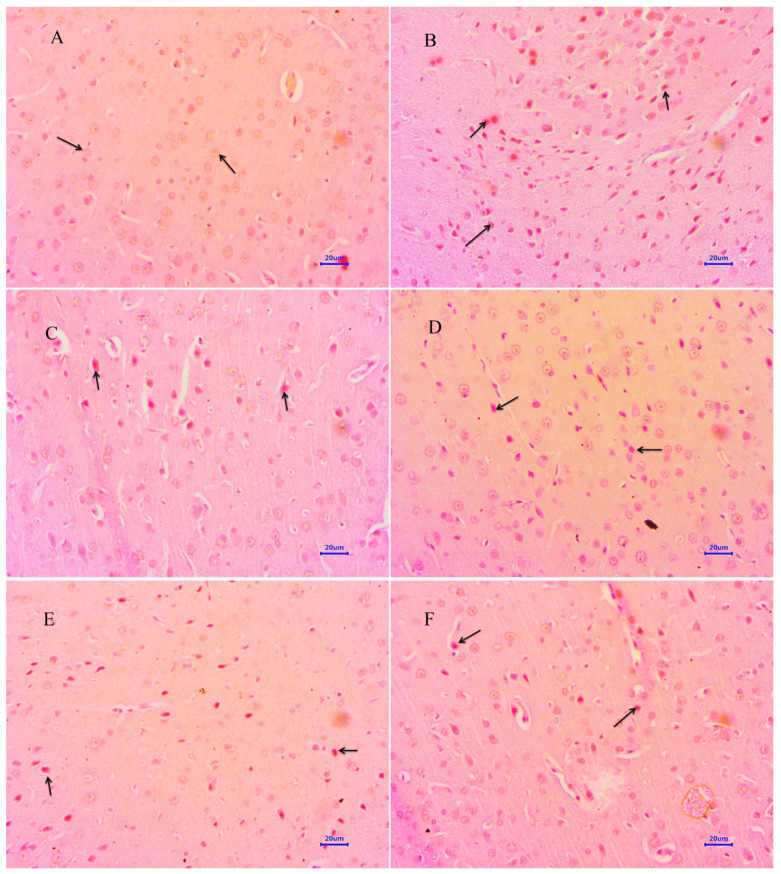
Effect of Aβ_(1–42),_ baicalein and baicalein with memantine on Congo red staining in cortex (400×). (**A**) Normal control: displaying normal histology, normal neuronal cells in parietal cortex (arrow). (**B**) Disease control: displaying multifocal moderate to severe deposition of deposition of β-amyloid (arrow) in parietal cortex. (**C**) Memantine 20 mg/kg: displaying moderate deposition of β-amyloid (arrow) in parietal cortex. (**D**) Baicalein (10 mg/kg): displaying multifocal mild to moderate deposition of β-amyloid (arrow) in parietal cortex. (**E**) Memantine + Baicalein (20 + 5 mg/kg): displaying multifocal mild deposition of β-amyloid (arrow) in parietal cortex. (**F**) Memantine + Baicalein (20 + 10 mg/kg): displaying mild deposition of β-amyloid (arrow) in parietal cortex.

Microscopic examination of the hippocampus and cortex section stained with Congo red from the normal control group did not show deposition of β-amyloid. However, the hippocampus and cortex from animals from the disease control group exhibited multifocal moderate to severe deposition of β-amyloid. Animals treated with memantine 20 mg/kg demonstrated multifocal mild to moderate deposition of β-amyloid in the hippocampus and cortex. The treatment of baicalein 10 mg/kg demonstrated multifocal mild to moderate deposition of β-amyloid in both the hippocampus and cortex of rat brain. Further, the combination treatment of baicalein with memantine at the dose of 20 + 5 mg/kg indicated multifocal mild deposition of β-amyloid in the hippocampus and cortex. Moreover, treatment of baicalein with memantine at the dose of 20 + 10 mg/kg showed mild β-amyloid deposition in the hippocampus and cortex of rat brain (Figure 8 and Figure 9).

### 3.5. Immunohistochemistry Assessment (IHC)

#### 3.5.1. Immunohistochemistry Assessment for β-amyloid 

**Figure 10:**
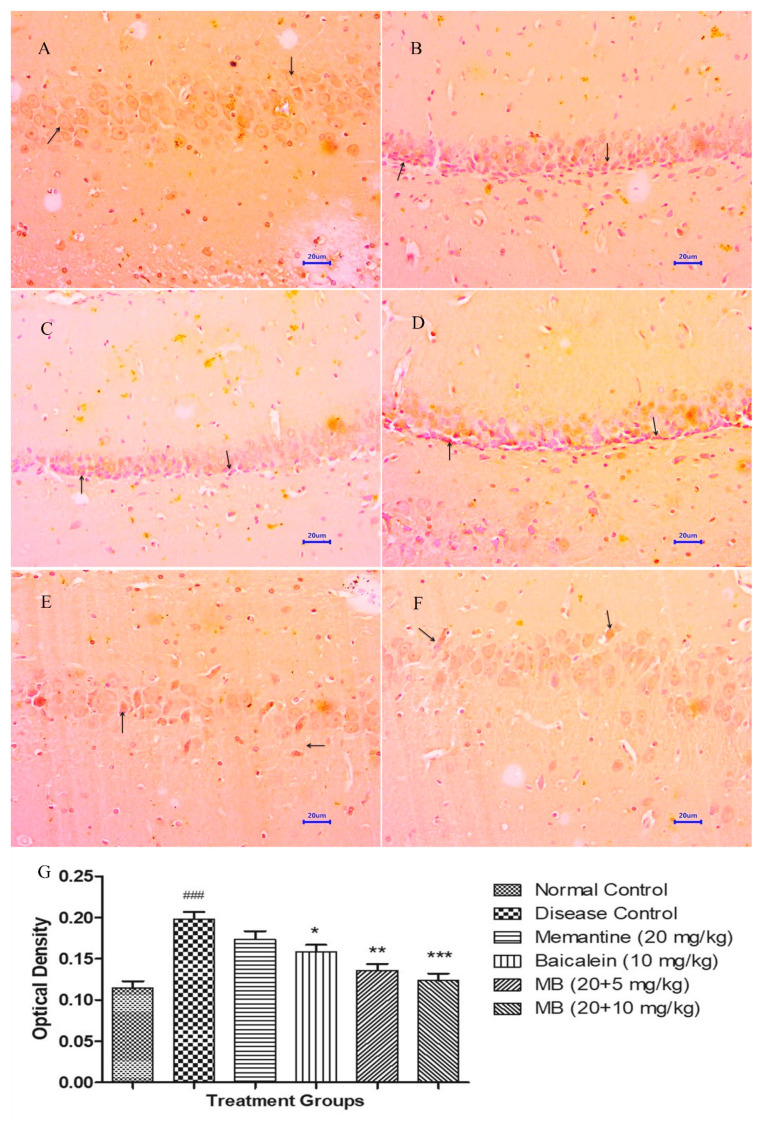
Effect of Aβ_(1–42),_ baicalein and baicalein with memantine on β-amyloid expression in hippocampus (400×). (**A**) Normal control: displaying morphologically normal neuronal cells (arrow) in hippocampus. (**B**) Disease control: displaying moderate to markedly enhanced β-amyloid deposition (arrow), exhibited by brown colouration in hippocampus. (**C**) Memantine 20 mg/kg: displaying mild β-amyloid deposition (arrow) exhibited by brown colouration in hippocampus. (**D**) Baicalein (10 mg/kg): displaying mild to moderate β-amyloid deposition (arrow) exhibited by brown colouration in hippocampus. (**E**) Memantine + Baicalein (20 + 5 mg/kg): displaying mild to moderate β-amyloid disposition (arrow) exhibited by brown colouration in hippocampus. (**F**) Memantine + Baicalein (20 + 10 mg/kg): displaying mild β-amyloid deposition (arrow) exhibited by brown colouration in hippocampus. (**G**) OD data for ICH. Data are represented as mean ± SEM, where ### *p* < 0.001 indicates comparison with normal control, whereas * *p* < 0.05, ** *p* < 0.01 and *** *p* < 0.001 indicates comparison with disease control group; SEM, Standard Error of Mean; MB: memantine + baicalein; IHC: Immunohistochemistry.

**Figure 11:**
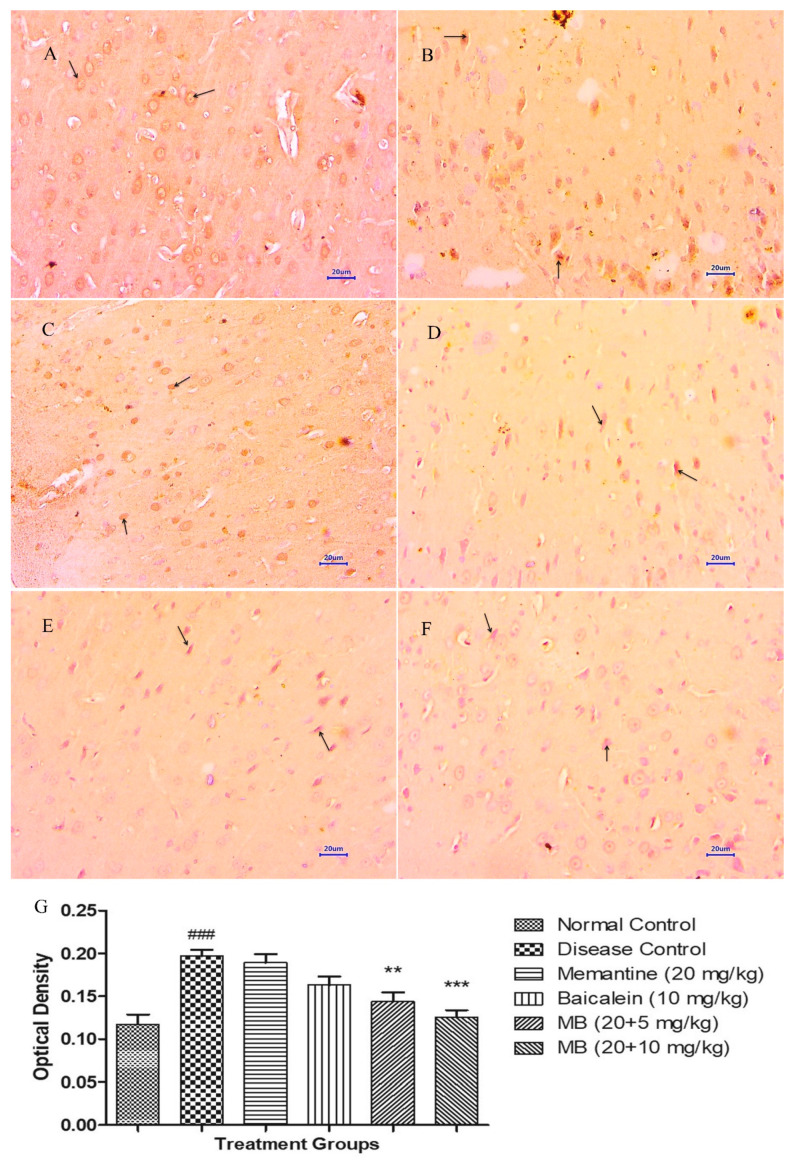
Effect of Aβ_(1–42),_ baicalein and baicalein with memantine on β-amyloid expression in cortex (400×). (**A**) Normal control: displaying morphologically normal neuronal cells (arrow) in cortex. (**B**) Disease control: displaying moderate to severe β-amyloid deposition (arrow), exhibited by brown colouration in cortex. (**C**) Memantine 20 mg/kg: displaying mild to moderate β-amyloid deposition (arrow) exhibited by brown colouration in cortex. (**D**) Baicalein (10 mg/kg): displaying mild to moderate β-amyloid deposition (arrow) exhibited by brown colouration in cortex. (**E**) Memantine + Baicalein (20 + 5 mg/kg): displaying mild β-amyloid disposition (arrow) exhibited by brown colouration in cortex. (**F**) Memantine + Baicalein (20 + 10 mg/kg): displaying mild β-amyloid deposition (arrow) exhibited by brown colouration in cortex. (**G**) OD data for ICH. Data are represented as mean ± SEM, where ### *p* < 0.001 indicates comparison with normal control, whereas ** *p* < 0.01 and *** *p* < 0.001 indicates comparison with disease control group; SEM, Standard Error of Mean; MB: memantine + baicalein; IHC: Immunohistochemistry.

Immunohistochemistry (IHC) evaluation of the hippocampus and cortex from the normal control group showed normal histology of neuronal cells without any deposition of Aβ. However, microscopic examination from the disease control group depicted moderate to markedly enhanced Aβ plaque deposits in both the hippocampus and cortex. Animals treated with memantine 20 mg/kg revealed mildly enhanced expression of Aβ exhibited by brown colouration in the hippocampus and cortex. Further, mild deposition of Aβ was visible in cortex, and mild to moderate deposition was visible in the hippocampus of animals treated with baicalein 10 mg/kg. The combination of baicalein with memantine at the dose of 20 + 5 mg/kg indicated mild expression of Aβ deposits in the cortex region and mild to moderate Aβ deposits in the hippocampus. Moreover, microscopic evaluation of the hippocampus and cortex from the treatment group of baicalein with memantine at a dose of 20 + 10 mg/kg demonstrated mild expression of Aβ deposits. (Figure 10 and Figure 11).

#### 3.5.2. Immunohistochemistry Assessment for BDNF 

**Figure 12:**
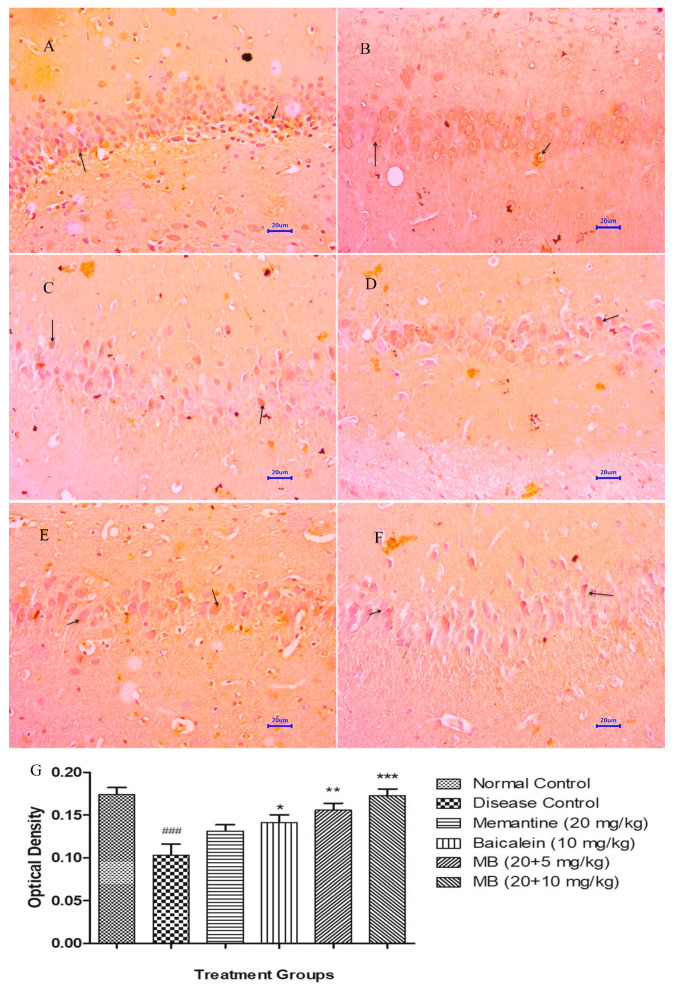
Effect of Aβ_(1–42),_ baicalein and baicalein with memantine on BDNF expression in hippocampus tissue (400×). (**A**) Normal control: displaying moderate expression of BDNF (arrow) revealed by brown colouration in hippocampus. (**B**) Disease control: displaying mild expression of BDNF (arrow), revealed by brown colouration in hippocampus. (**C**) Memantine 20 mg/kg: displaying mild expression of BDNF (arrow), revealed by brown colouration in hippocampus. (**D**) Baicalein (10 mg/kg): displaying mild expression of BDNF (arrow) revealed by brown colouration in hippocampus. (**E**) Memantine + Baicalein (20 + 5 mg/kg): displaying mild to moderate expression of BDNF (arrow), revealed by brown colouration in hippocampus. (**F**) Memantine + Baicalein (20 + 10 mg/kg): displaying moderate expression of BDNF (arrow), revealed by brown colouration in hippocampus. (**G**) OD data for ICH. Data are represented as mean ± SEM, where ### *p* < 0.001 indicates comparison with normal control, whereas * *p* < 0.05, ** *p* < 0.01 and *** *p* < 0.001 indicates comparison with disease control group; SEM, Standard Error of Mean; MB: memantine + baicalein.

**Figure 13:**
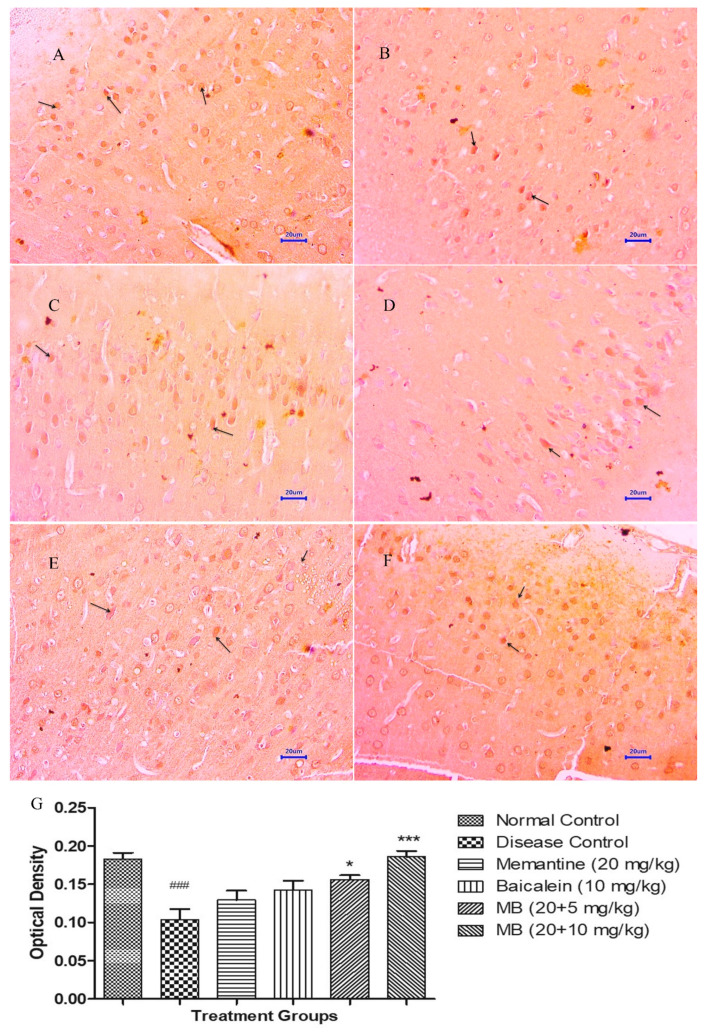
Effect of Aβ_(1–42),_ baicalein and baicalein with memantine on BDNF expression in hippocampus tissue (400×). (**A**) Normal control: displaying moderate expression of BDNF (arrow) revealed by brown colouration in cortex. (**B**) Disease control: displaying mild expression of BDNF (arrow), revealed by brown colouration in cortex. (**C**) Memantine 20 mg/kg: displaying mild expression of BDNF (arrow), revealed by brown colouration in cortex. (**D**) Baicalein (10 mg/kg): displaying mild expression of BDNF (arrow) revealed by brown colouration in cortex. (**E**) Memantine + Baicalein (20 + 5 mg/kg): displaying mild to moderate expression of BDNF (arrow), revealed by brown colouration in cortex. (**F**) Memantine + Baicalein (20 + 10 mg/kg): displaying moderate expression of BDNF (arrow), revealed by brown colouration in cortex. (**G**) OD data for ICH, Data are represented as mean ± SEM, where ### *p* < 0.001 indicates comparison with normal control, whereas * *p* < 0.05 and *** *p* < 0.001 indicates comparison with disease control group; SEM, Standard Error of Mean; MB: memantine + baicalein.

The microscopic evaluations of the hippocampus and cortex demonstrated decreased expression of BDNF in animals from the disease control group, whereas the normal control group demonstrated moderately enhanced expression of BDNF in both the hippocampus and cortex. The treatment of memantine (20 mg/kg) alone and baicalein (10 mg/kg) alone indicated mildly enhanced BDNF expression in both the hippocampus and cortex. Furthermore, the combination treatment of baicalein and memantine at the dose of 20 + 5 mg/kg augmented neuronal health, which can be confirmed by the brown colouration of moderate expression of BDNF. Similarly the combination treatment of baicalein with memantine at a dose of 20 + 10 mg/kg also demonstrated significant enhancement in neuronal survival and plasticity depicted by the dark brown colouration of moderate BDNF expression in both the hippocampus and cortex [34]. The OD data plotted against treatment group also demonstrated the highest OD for the combination treatment of baicalein and memantine at the dose of 20 + 10 mg/kg when compared with the disease control group (Figure 12 and Figure 13).

## 4. Discussion

The most critical pathological features of Alzheimer’s disease are the formation of extracellular β-amyloid plaque and intracellular NFTs formed by hyper-phosphorylated tau protein [35]. In this study, Aβ_(1–42)_ was microinjected into the adult rat brain using the stereotaxic method, resulting in neurodegeneration [36]. To evaluate the effect of baicalein with memantine, various behavioural models such as Actophotometer, MWM, EPM and PA were studied. Furthermore, oxidative stress parameters, cholinesterase inhibition activity, histopathological and immunohistochemistry evaluations were performed [37]. 

The study results indicated significant impairment in the locomotor activity of the disease control animals. However, the combination treatment of baicalein with memantine at a dose of 20 + 10 mg/kg demonstrated a significant enhancement in the locomotor activity in comparison with the disease control group. In the MWM test, baicalein with memantine combination at a dose of 20 + 10 mg/kg showed extensive improvements in memory and learning skills when compared with the diseases control group. Moreover, a decrease in the transfer latency of the disease control group and significant increase in the animals treated with the combination treatment of baicalein and memantine at the dose of 20 + 10 mg/kg was observed in EPM. In the PA test, a decline in memory retention was observed in disease control group. On the contrary, a significant increase in memory retention was observed in the group treated with the baicalein and memantine combination at a dose of 20 + 10 mg/kg. 

Aβ has been identified as a causative source of oxidative stress, cellular damage and synaptic loss, resulting in cognitive and memory dysfunction [38]. The present study results from the behavioural and memory assessments indicated that memory, learning and cognitive characteristics in the animals injected with Aβ_(1–42)_ declined over a period of time during the study, whereas a significant improvement was observed in the treatment group of baicalein with memantine, indicating a significant ameliorating effect of combination treatment in Aβ-induced neurodegeneration.

Administration of Aβ through intracerebroventricular (i.c.v.) injection for pathophysiological traits of neurodegeneration is more relevant to sporadic AD. Clinical symptoms such as oxidative stress, neuroinflammation and cholinergic dysfunction were observed in this animal model [39], allowing us to study the effect of treatment with baicalein and memantine. 

Oxidative stress in the AD brain induced by Aβ_(1–42)_ is manifested by decreasing antioxidative enzymes, increased protein oxidation and lipid peroxidation [40]. Mitochondria are the key organelle responsible for the generation of ROS (reactive oxygen species) and also provide ATP to the host cell by oxidative phosphorylation. To maintain ionic gradients and neurotransmission, neurons need a significant amount of ATP. As neurons cannot store ATP, unlike other cells, the normal function of mitochondria in neuronal cells becomes very crucial for a continuous supply of energy. 

Furthermore, neurons are extremely vulnerable to ROS. It is known that Aβ reduces mitochondrial superoxide dismutase, which is responsible for protecting peroxidative damage [41]. It has also been reported that mitochondrial dysfunction is responsible for alterations in amyloid precursor protein (APP) metabolism, resulting in further intra-neuronal accumulation of Aβ, leading to neurodegeneration [42,43]. The significant decrease in MDA and the increase in SOD, catalase and GSH in the disease control group demonstrated the oxidative damages due to Aβ_(1–42)._

The anti-oxidant properties of baicalein are mediated mainly through the prevention of mitochondrial dysfunction by reducing a loss in matrix metalloproteinases (MMP) and activating nuclear factor erythroid 2-related factor (Nrf2) [15]. Our study results demonstrated that at a dose of 20 + 10 mg/kg, baicalein with memantine significantly decrease MDA levels and increase SOD, catalase and GSH, which accentuates the anti-oxidant properties of baicalein with memantine.

The objective of AD treatment is to re-establish the cholinergic functions that are disturbed due to the dysfunction of synapses, leading to memory impairment and cognitive abnormalities. Various enzymatic studies and SAR have indicated AChE-inhibitory activity of baicalein [44,45,46]. The study results further established that treatment of baicalein with memantine at a dose of 20 + 10 mg/kg significantly reduced the AChE activity in the hippocampus and cortex region, which supports an improvement in memory and cognition. 

The histopathological evaluation of H&E and Congo red stain was performed to assess the extent of neurodegeneration and Aβ plaque formation in the present study [35,36]. Histopathological studies in the normal control group by the H&E-stained brain revealed no lesions of pathological significance, whereas the disease control group showed multifocal moderate neurodegeneration in the hippocampus and cortex. However, animals treated with the baicalein and memantine combination at a dose of 20 + 10 mg/kg showed focal minimal neuronal degeneration in the hippocampus and cortex, which establishes the role of the combination treatment of baicalein with memantine in the inhibition of neurodegeneration caused by Aβ. Further, the Congo-red-stained sections of the brain revealed significant extent of Aβ plaque deposition in hippocampus and cortex in animals from the disease control group. However, animals treated with baicalein and memantine at 25 + 10 mg/kg showed focal minimal to mild deposition of Aβ in both the hippocampus and cortex, which highlight anti-amyloid plaque activity of combination therapy of baicalein with memantine. 

Immunohistochemical (IHC) evaluations also confirmed the inhibition of β-Amyloid plaque in the animal group treated with baicalein and memantine at a dose of 20 + 10 mg/kg. Further, expression of BDNF which is an indicator of neuronal health, plays a crucial role in both maintaining synaptic transmission and plasticity. BDNF can also modulate several signalling pathways that are associated with learning and memory. The BDNF signalling pathways have also been linked to neuroinflammation, Aβ plaque formation, phosphorylation of tau proteins and neuronal apoptosis [10,47]. In the present study, ICH evaluation of the disease control group showed a BDNF deficiency. However, the evaluation from the group treated with baicalein with memantine showed an enhancement in the expression of BDNF in the hippocampus and cortex, confirming its neuroprotective effect. 

## 5. Conclusions

Alzheimer’s disease is a multi-factorial disease and therefore, demands a novel multi-target drug therapy.

The results from the present study demonstrated the multi-therapeutic properties of baicalein in combination with memantine. The results indicate this therapy as an anti-oxidant, AChE-inhibitory, memory- and learning-enhancing, Aβ-plaque-reducing, and BDNF-expression-enhancing agent; therefore, it has a combined neuroprotective effect against Aβ-induced AD in albino Wistar rats. 

The study outcomes clearly indicate that the effect of baicalein in combination with memantine is greater than the effect of baicalein and memantine when administered alone. Hence, baicalein has an additive effect in combination therapy with memantine. 

In conclusion, the results of the study propose that baicalein and its combination with memantine could be a promising approach for the management of AD.

## Figures and Tables

**Table 1:** Effect of Aβ_(1–42)_, baicalein and baicalein with memantine on oxidative stress parameters in the rat brain hippocampus.

Groups	MDA (nmol/mg of Protein)	SOD (µmol/mg of Protein)	CAT (nmol of H_2_O_2_ Decomposed/ min/mg of Protein)	GSH (µmol/mg of Protein)
Normal control	4.10 ± 0.31	8.65 ± 0.48	9.88 ± 0.46	11.00 ± 0.36
Disease control	8.01 ± 0.63 ^###^	4.84 ± 0.28 ^###^	4.03 ± 0.60 ^###^	4.95 ± 0.36 ^###^
Memantine (20 mg/kg)	6.85 ± 0.30	6.09 ± 0.41	4.97 ± 0.36	5.95 ± 0.57
Baicalein (10 mg/kg)	6.22 ± 0.37 *	6.63 ± 0.53	6.25 ± 0.52 *	6.84 ± 0.58 *
Memantine (20 mg/kg) + Baicalein (5 mg/kg)	5.84 ± 0.42 **	7.09 ± 0.57 *	7.40 ± 0.51 ***^/@^	8.75 ± 0.37 ***^/@@@^
Memantine (20 mg/kg) + Baicalein (10 mg/kg)	4.96 ± 0.36 ***^/@^	7.88 ± 0.69 ***^/@@^	8.73 ± 0.62 ***^/@@@^	9.76 ± 0.41 ***^/@@@^

Data are represented as mean ± SEM (n = 6), ### *p* < 0.001 indicates comparison with normal control, * *p* < 0.05, ** *p* < 0.01 and *** *p* < 0.001 indicates comparison with disease control group, whereas @ *p* < 0.05, @@ *p* < 0.01 and @@@ *p* < 0.001 indicates comparison with memantine (20 mg/kg) group. SEM: Standard Error of Mean.

**Table 2:** Effect of Aβ_(1–42)_, baicalein and baicalein with memantine on oxidative stress parameters in the rat brain cortex.

Groups	MDA (nmol/mg of Protein)	SOD (µmol/mg of Protein)	CAT (nmol of H_2_O_2_ Decomposed/ min/mg of Protein)	GSH (µmol/mg of Protein)
Normal control	4.16 ± 0.35	8.10 ± 0.46	8.71 ± 0.41	10.31 ± 0.67
Disease control	7.49 ± 0.43 ^###^	4.41 ± 0.28 ^###^	4.35 ± 0.34 ^###^	5.06 ± 0.39 ^###^
Memantine (20 mg/kg)	6.95 ± 0.42	5.25 ± 0.37	5.52 ± 0.30	6.36 ± 0.57
Baicalein (10 mg/kg)	5.37 ± 0.54 **^/@^	6.96 ± 0.29 ***^/@^	6.13 ± 0.29 **	7.20 ± 0.54 *
Memantine (20 mg/kg) + Baicalein (5 mg/kg)	4.91 ± 0.33 ***^/@@^	7.46 ± 0.45 ***^/@@^	7.32 ± 0.45 ***^/@@^	8.02 ± 0.53 **
Memantine (20 mg/kg) + Baicalein (10 mg/kg)	4.55 ± 0.38 ***^/@@^	7.74 ± 0.48 ***^/@@@^	8.29 ± 0.43 ***^/@@@^	9.11 ± 0.45 ***^/@^

Data are represented as mean ± SEM (n = 6), ### *p* < 0.001 indicates comparison with normal control, * *p* < 0.05, ** *p* < 0.01 and *** *p* < 0.001 indicates comparison with disease control group, whereas @ *p* < 0.05, @@ *p* < 0.01 and @@@ *p* < 0.001 indicates comparison with memantine (20 mg/kg) group. SEM: Standard Error of Mean.

## Data Availability

Authors agree to make supporting data available on request.
